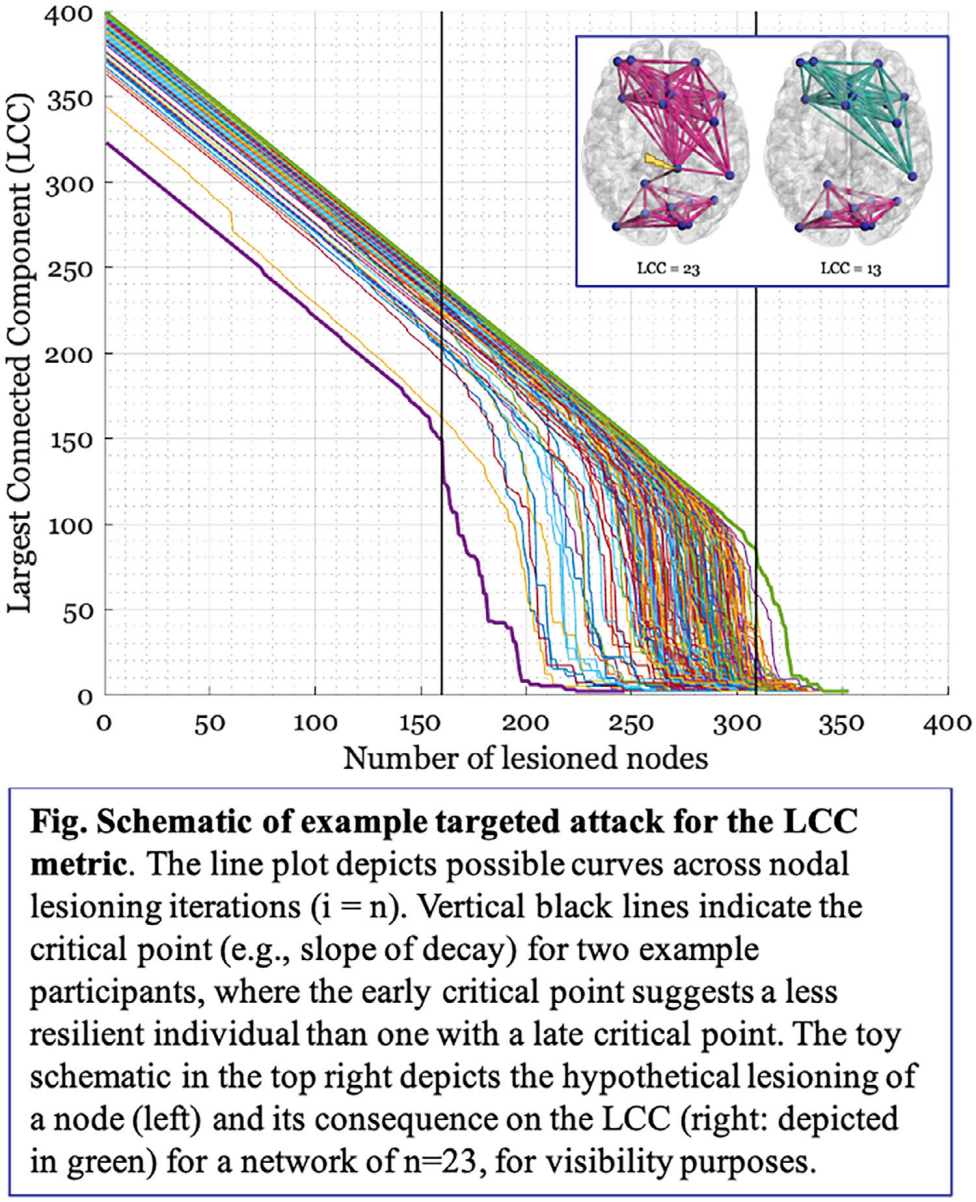# Brain Resilience to Targeted Attack of Resting BOLD Networks as a Measure of Cognitive Reserve

**DOI:** 10.1002/alz.087406

**Published:** 2025-01-09

**Authors:** Georgette Argiris, Yaakov Stern, Christian G Habeck

**Affiliations:** ^1^ Columbia University Irving Medical Center, New York, NY USA

## Abstract

**Background:**

Recent advancements in connectome analyses allow for more fine‐grained measurements of brain network integrity. One measure of integrity is resilience, or the capacity of the network to retain functionality when confronted with endogenous or exogenous perturbations that result in damage or error. We assessed the impact of individual differences in the resilience of resting BOLD connectivity on the relationship between cognitive and brain changes in a lifespan cohort of cognitively healthy adults over a 5‐year period.

**Method:**

One hundred twenty‐six cognitively healthy participants from the Reference Ability Neural Network (RANN) longitudinal lifespan cohort (age 20‐80 years) underwent resting‐state fMRI to measure functional connectivity and an out‐of‐scanner neuropsychological battery at baseline and five‐year follow‐up. Undirected weighted adjacency matrices were generated from Schaefer et al. (2018) 400 parcellation atlas. As a measure of whole‐brain network resilience, we adopted a targeted attack approach, whereby nodes are sequentially removed from the connectome in order of nodal strength. At each iteration of attack, nodal strength is recalculated based on the effect of prior lesioning and the largest connected component (LCC) is measured. We inferred that more resilient individuals will sustain larger LCCs over longer iterations of lesioning before decay in LCC becomes evident, with resilience operationalized as the iteration of steepest slope in LCC.

**Result:**

We tested whether our operationalization of brain resilience (BR) moderated the effect of brain integrity (i.e., cortical thickness; CT) on out‐of‐scanner neuropsychological test performance across four domains of cognition in the context of longitudinal change (∆) over time. After accounting for baseline differences in change variables and adjusting for the demographic factors of Age, Sex, NART IQ, and Education, we observed a significant negative interaction between ∆CT and ∆BR on ΔCognition for the Fluid Reasoning domain. That is, individuals with increased brain resilience over time were less sensitive to the effect of changes in cortical thickness on changes in cognition.

**Conclusion:**

Our finding supports evidence for targeted attack as a measure of cognitive reserve, where higher brain network resilience may have permitted individuals with reduced brain integrity to better cope with structural loss and enhance preservation of cognitive function.